# Survival Analysis of Breast Cancer Patients in Texas Using Classical and Machine Learning Methods

**DOI:** 10.7759/cureus.96250

**Published:** 2025-11-06

**Authors:** Sidketa I Fofana, Tamer Oraby, Salique H Shaham, Everardo Cobos, Manish K Tripathi

**Affiliations:** 1 School of Mathematical and Statistical Sciences, The University of Texas Rio Grande Valley, Edinburg, USA; 2 Medicine and Oncology Integrated Service Unit (ISU), The University of Texas Rio Grande Valley, McAllen, USA; 3 South Texas Center of Excellence in Cancer Research, The University of Texas Rio Grande Valley, McAllen, USA; 4 Medicine and Oncology Integrated Service Unit (ISU), The University of Texas Rio Grande Valley, Edinburg, USA

**Keywords:** breast cancer, cox proportional hazards regression, kaplan‒meier curves, log-rank test, mahalanobis matching distance, racial equity, random survival forest

## Abstract

Background

Breast cancer is considered one of the most common cancers in women worldwide. In this study, we used an 11-year cohort of malignant breast cancer survival data in Texas to investigate the factors that might explain why some breast cancer patients live longer than others.

Methods

We performed standard survival analyses, including generating Kaplan‒Meier survival curves, using the log-rank test, and applying Cox proportional hazards regression to identify the unique features of breast cancer patients and determine the main factors influencing long-term survival. We also conducted a Random Survival Forest analysis for classification and prediction. Finally, we used Mahalanobis matching distance to estimate the average extended lifetime of patients in different groups.

Results

Our study found that stage, laterality, age, grade, subtypes, progesterone receptor status, the primary site of cancer, patient race, median household income, chemotherapy, systemic therapy, and surgery are significant factors, with stage being the most critical for predicting survival in breast cancer patients. Compared to individuals with localized-stage breast cancer, those with distant-stage cancer had a higher hazard ratio (15.869). We also observed that patients diagnosed at the localized stage had an average survival of approximately 67.34 months, compared to those diagnosed at the distant stage.

Conclusion

Policymakers should focus on promoting early diagnosis and screening, while also providing additional support to the elderly and those in disadvantaged circumstances.

## Introduction

According to the American Cancer Society (ACS) 2025 statistics and the Centers for Disease Control and Prevention (CDC), breast cancer is the second most common cancer among women in the United States of America (USA), after skin cancer, across all races and ethnicities. An estimated 319,750 women will be diagnosed with breast cancer in 2025, and 42,680 women are expected to die from it in the same year [1,2]. In the USA, one in every eight women is expected to be diagnosed with invasive breast cancer during her lifetime, according to the National Cancer Institute [3]. Breast cancer national expenditures in the USA were estimated to be $29.8 billion in 2020 [4]. Furthermore, when comparing treatment costs for breast cancer by tumor stage using a sample size of 8,360 women in the USA, it is reported that the average costs per patient allowed by the insurance in the year after diagnosis were $60,637 for stage 0, $82,121 for stages I and II, $129,387 for stage III, and $134,682 for stage IV [5]. Therefore, breast cancer is a burden on taxpayers, a public health concern, and is linked to significant productivity loss.

In this study, we focus on the state of Texas. Breast cancer is the most diagnosed cancer among women in Texas and the second leading cause of cancer-related death [6]. In 2022, following lung and bronchus cancer, the Department of State Health Services (DSHS) estimated that 19,921 women were diagnosed with breast cancer in Texas, and a total of 3,415 women are expected to die from it.

The most common types of breast cancer are invasive ductal carcinoma and invasive lobular carcinoma [7]. The seed and soil theory of metastasis, proposed by Stephen Paget in 1889, compares the spread of cancer cells to seeding fertile land [8]. Metastatic tropisms refer to cancer cells' tendencies to favor specific organs or tissues when spreading. Several factors influence these preferences, including the interactions between cancer cells and the microenvironment of the target organ. For example, breast cancer cells tend to metastasize to the brain, bones, and lungs [9], while lung cancer cells often spread to the brain or other lung tissues [10]. Research has provided insights into the molecular mechanisms behind metastatic tropisms and the interactions between circulating tumor cells and the microenvironment of target organs [11]. Understanding the intricacies of metastatic tropisms and the interactions between malignant cells and the microenvironment of target organs is an active area of research in oncology. These insights hold promise for developing targeted therapies that disrupt the mechanisms driving metastasis, thereby potentially enhancing outcomes for cancer patients.

We used the 11-year cohort study of breast cancer survival data in Texas to identify health and socioeconomic disparities that might explain why some breast cancer patients live longer than others [12]. Understanding these factors helps us pinpoint the highest-risk cancer survivors. This allows us to make effective and resourceful recommendations to help new patients live longer, better lives and reduce breast cancer's burden on society. Since over 99% of breast cancer patients are women, we focused on the 14,500 women diagnosed with malignant breast cancer in 2010 from the Surveillance, Epidemiology, and End Results (SEER) database, followed up to 2021 in Texas.

We used various statistical methods to better understand the main factors affecting breast cancer survival and the disparities among them. We acknowledge that an earlier version of this article was previously posted on Research Square.

The remaining sections of the work are organized as follows: background information and review of related literature, materials and methods, results, discussion, and conclusion.

Background and literature review

The main risk factors for breast cancer include advanced age, genetic mutations, early onset of menstruation, late or no pregnancies, starting menopause after age 55, lack of physical activity, being overweight or obese after menopause, having dense breasts, using hormonal therapy, taking oral contraceptives, having a family history of breast cancer, drinking alcohol, smoking cigarettes, and women from high socioeconomic backgrounds [13]. Therefore, preventing breast cancer can be challenging.

A mammogram is a screening tool that doctors use to detect breast cancer early, sometimes up to three years before any physical symptoms appear. However, mammography is not perfect because it can sometimes produce diagnostic errors [14]. Recently, a new type of mammogram for breast cancer detection called a 3D mammogram using tomosynthesis has been developed. In January 2018, Texas House Bill 1036 required all insurance companies to cover 3D mammograms for Texas residents. There is a continued need to explore improved screening and diagnostic tools in the future, as these tools will aid in early detection.

Since breast cancer can be a recurrent or chronic disease, the main goal for survivors is to maximize their survival time. Therefore, it is essential to understand why some survivors live longer than others. Additionally, cancer mortality is associated with significant productivity losses, as demonstrated by Bradley et al. [15], who studied the productivity costs of cancer mortality in the U.S. from 2000 to 2020. Their study shows that adding imputed earnings lost from informal caregiving increased the total productivity cost in the United States from $232.4 billion in 2000 to $308 billion in 2020.

Breast cancer survival and race

Disparities in breast cancer mortality from 1979 to 2010 among African American women aged 20 to 49 have been examined [16]. They found that disease-specific mortality rates decreased over time for some conditions. However, mortality rates consistently remained higher for Black women with breast cancer, cervical cancer, colorectal cancer, ischemic heart disease, or stroke. The mortality rate ratio increased for breast cancer patients throughout the study period. The annual mortality rate ratio for Black women compared to White women was 1.36 in 1979 and rose to 2.00 in 2010. Additionally, racial differences in breast cancer mortality based on stage at diagnosis, since mammography became available, continue to persist [17]. In conclusion, even during the era of mammography, racial disparities at diagnosis remain.

Investigating the reasons for ethnic inequalities in breast cancer survival in New Zealand revealed that inequalities persisted even after adjusting for estrogen receptor, progesterone receptor, and human epidermal growth factor receptor 2 (ER/PR/HER2) subtype variables. However, adjusting for access-to-care factors (such as extent and size) eliminated the ethnic inequalities in excess mortality [18]. The authors concluded that ethnic disparities in breast cancer survival in New Zealand are more likely due to deprivation and unequal access to healthcare rather than differences in breast cancer subtypes. In this study, we control for race and ethnicity to determine whether disparities in survival time exist due to race, considering Texas's diverse racial and ethnic population.

Age at diagnosis and survival of patients with breast cancer

Age at diagnosis plays a crucial role in breast cancer survival [19], as shown by the relationship between age at diagnosis and relative survival in 57,068 women in Sweden diagnosed with breast cancer between 1960 and 1978. Their findings indicate that women aged 45 to 49 have the best prognosis, with a relative survival rate higher than that of the youngest patients. They also observed that relative survival significantly decreased after age 49, especially in women aged 50 to 59 and those older than 75. The authors concluded that the long-term annual mortality rate from breast cancer approached 1 to 2% at premenopausal ages but exceeded 5% throughout the observation period in the oldest age group.

Additionally, changes in physical and psychosocial functions before and after a breast cancer diagnosis were observed across different age groups [20]. They concluded that, compared to women aged 40 without breast cancer, women with breast cancer experienced significant declines in function. In this study, we examined whether age at diagnosis remains an important factor for survival among breast cancer patients in Texas.

Comorbidity and breast cancer survival

The presence of comorbidities, such as diabetes and asthma, is linked to early death from breast cancer. Racial differences and the effect of comorbidities on breast cancer-specific survival have been studied [21]. The study involved a retrospective cohort of 68,090 women aged 66 and older diagnosed with stages I-III breast cancer in the U.S. from 1994 to 2004. The findings indicate that diabetes without complications is associated with a significantly higher risk of breast cancer-specific death among white patients, with aggressive tumor features explaining part of this effect. Additionally, obesity, smoking, alcohol use, and other comorbidities negatively affect survival rates in breast cancer patients.

Obesity and breast cancer 

Recent studies agree that obesity influences both the risk of developing breast cancer and survival rates afterward. The connections between obesity, physical activity, and breast cancer survival among older survivors in the Cancer Prevention Study II Nutrition Cohort from 1992 to 2013 have been documented [21]. Researchers concluded that a higher BMI, either before or after diagnosis, was linked to an increased risk of breast cancer-specific death in older patients, regardless of comorbidities and stage at diagnosis [22].

Similarly, in a study examining the relationship between obesity and quality of life (QOL) among Hispanic and non-Hispanic white breast cancer survivors, a 12- to 15-year follow-up study of breast cancer patients, survivors, and controls from New Mexico (n = 451) was conducted [23]. They found that baseline obesity was signiﬁcantly associated with decreased mental health among survivors but not among controls. Obesity at baseline and follow-up was significantly associated with decreased physical health among survivors (baseline b=-10.51, p-value=0.004; follow-up b=-7.16, p-value=0.02) and controls (baseline b=-11.07, p-value=0.001; follow-up b=-5.18, p-value=0.04). No significant interactions between ethnicity and BMI were observed.

Stage and grade at diagnosis and breast cancer survival

The stage and grade of cancer at diagnosis significantly influence survivorship duration and the treatment a patient receives. Staging assesses tumor size, whether it has spread, and the extent of spread. Knowing the cancer stage helps doctors predict outcomes and tailor treatment plans for each individual. We controlled for the stage of diagnosis in this study. Besides tumor stage, tumor grade is a crucial factor that influences both survival rates and treatment options. The tumor grade describes how the cancer cells appear and provides insight into how quickly the cancer may grow and spread.

Neighborhood poverty level, household median income, and breast cancer survival status

Access to quality care is essential for breast cancer prognosis. Some costs are linked to caring for survivors, such as the expense of healthy food needed to stay active, including gym memberships. Therefore, financial status is important, and to account for it, I consider the neighborhood poverty level based on the census tract of the diagnosis address and the patient's household median income. We also examined the survivors' location, such as whether they reside in metropolitan, urban, or rural areas, as this can impact the type of care they receive. For example, there are more cancer hospitals in large cities than in rural areas, and healthier food stores tend to be in cities rather than rural regions. All these factors can significantly influence breast cancer patients' prognosis.

## Materials and methods

In this retrospective cohort study from Texas, data for breast cancer patients were obtained from the Surveillance, Epidemiology, and End Results (SEER) Program (www.seer.cancer.gov) SEER*Stat Database: Incidence - SEER Research Plus Limited-Field Data, 22 Registries (excluding Illinois and Massachusetts), Nov 2023 submission (2000-2021) - Linked to County Attributes - Time Dependent (1990-2022) Income/Rurality, 1969-2022 Counties, National Cancer Institute, Division of Cancer Control and Population Science (DCCPS), Surveillance Research Program, released April 2024, based on the November 2023 submission. The study was determined to be ‘exempt’ under the basic HHS Policy for Protection of Human Research Subjects, 45 CFR 46.104(d), with IRB-24-0193 at the University of Texas Rio Grande Valley (UTRGV).

We first selected all women diagnosed with malignant breast cancer recorded by the SEER Program in 2010 and 2011 who were followed up to 2021. According to SEER, 28,881 women fit this description. We will perform a breast cancer-specific survival analysis, including an examination of time-to-event data using the survival months for each patient. We excluded 299 patients from the dataset who had unknown survival months. To evaluate breast cancer-related death, we will use the variable SEER Cause-Specific Death. We excluded 123 patients with missing or unknown causes of death and 118 patients with missing information about subtypes, estrogen receptor, progesterone receptor, and HER2 receptor. Patients still alive at the last follow-up or who died from causes other than breast cancer are right-censored. Finally, the event of interest is death attributed to breast cancer. After removing 1.87% (540 patients) due to missing information, our final dataset included 28,341 patients.

Statistical analyses and visualization were conducted using the statistical software SAS (SAS Institute Inc., Cary, NC), specifically the Proc lifetest procedure for Kaplan-Meier survival curves and log-rank tests [24] and the Proc phreg procedure for stratified and non-stratified Cox proportional hazard regression analyses [25,26]. We analyzed the random survival forests on the statistical software R via the randomForestSRC package. The Random Survival Forest is an extension of the Random Forests method for analyzing right-censored survival data. It performs well with high-dimensional data and allows for complicated interaction effects between covariates without requiring proportional risks [27]. For the causal inference, we use the MatchIt package. Matching is a valuable nonparametric method for improving causal inferences, especially in observational studies when treatment and control groups are not managed by the researcher. It is gaining popularity among applied researchers. In this study, we will use Mahalanobis distance matching, a widely used and traditional matching approach. The Mahalanobis distance measures the distance between two N-dimensional points, adjusted by the statistical variation in each component of the points [28].

## Results

Data descriptions

We begin with descriptive statistics to examine the various variables that characterize breast cancer patients. These variables include age, stage, grade, subtypes, estrogen receptor, progesterone receptor, HER2 receptor, the primary site of cancer, laterality, patient race, the total number of cancers each patient has, median household income, and the type of place where the patient lived, as well as the therapies received such as chemotherapy, radiation, systemic therapy, and surgery.

Summaries of the variables for breast cancer patients diagnosed in Texas in 2010 are provided in Table 1.

**Table 1:** Exploratory descriptive analysis of the characteristics of breast cancer survivors who were diagnosed in 2010 and 2011 in Texas. HR: Hormone Receptor; HER 2: Human Estrogen Receptor; NA: Not Applicable

Variables	Frequency/ Percentage	White non-Hispanic	Black non-Hispanic	Hispanic (all races)	Other
Race					
White non-Hispanic	18,222 (64.30%)	18,222	NA	NA	NA
Black non-Hispanic	3,429 (12.10%)	NA	3,429	NA	NA
Hispanic (all Race)	5,875 (20.73%)	NA	NA	5,875	NA
Other	815 (2.88%)	NA	NA	NA	815
Age (Years)					
between 15 and 44	3585 (12.65%)	9.26%	16.74%	19.69%	20.37%
between 45 and 54	6121 (21.60%)	19.19%	26.54%	24.99%	30.18%
between 55 and 64	7407 (26.14%)	26.63%	25.81%	24.89%	25.52%
between 65 and 74	6194 (21.86%)	24.26%	17.56%	17.70%	16.20%
75 and over	5034 (17.76%)	20.66%	13.36%	12.73%	7.73%
Therapy					
Chemotherapy (yes)	9427 (33.26%)	29.22%	42.02%	39.91%	38.90%
Chemotherapy (No/Unknown)	18914 (66.74%)	70.78%	57.98%	60.09%	61.10%
Radiation (yes)	6301 (22.23%)	22.84%	22.78%	19.93%	23.07%
Radiation (No/Unknown)	22040 (77.77%)	77.16%	77.22%	80.07%	76.93%
Systemic Therapy Surgery (yes)	12054 (42.53%)	41.07%	45%	44.68%	49.45%
Systemic Therapy Surgery (No/Unknown)	16287 (57.47%)	58.93%	55%	55.32%	50.55%
Total in situ\ malignant					
Total in situ \malignant =1	22222 (78.41%)	76.70%	79.99%	81.86%	85.03%
Total in situ \malignant =2	5094 (17.97%)	19.13%	17.44%	15.34%	13.37%
Total in situ\ malignant ≥3	1025 (3.62%)	4.17%	2.57%	2.81%	1.60%
Subtypes					
HR+/HER2+	2481 (8.75%)	8.34%	9.24%	9.70%	9.20%
HR+/HER2-	13946 (49.21%)	51.34%	41.88%	46.60%	51.04%
HR-/HER2+	960 (3.39%)	2.93%	3.67%	4.41%	5.15%
HR-/HER2-	2783 (9.82%)	7.90%	18.08%	11.34%	6.99%
Unknown	8171 (28.83%)	29.49%	27.12%	27.95%	27.61%
Estrogen Receptor					
Borderline/Unknown	5278 (18.62%)	19.12%	17.26%	17.82%	19.02%
Negative	4501 (15.88%)	13.17%	25.69%	18.81%	14.23%
Positive	18562 (65.50%)	67.71%	57.04%	63.37%	66.75%
Progesterone Receptor					
Borderline/Unknown	5405 (19.07%)	19.59%	17.41%	18.38%	19.51%
Negative	6686 (23.59%)	20.57%	34.47%	26.89%	21.60%
Positive	16250 (57.34%)	59.85%	48.12%	54.72%	58.90%
Derived HER 2					
Borderline/Unknown	8018 (28.29%)	28.95%	26.60%	27.39%	27.12%
Negative	16836 (59.41%)	59.61%	60.34%	58.35%	58.40%
Positive	3487 (12.30%)	11.43%	13.07%	14.26%	14.48%
Grade					
Grade I	2649 (9.35%)	10.59%	6.24%	7.37%	8.96%
Grade II	4931 (17.40%)	18.04%	15.46%	16.63%	16.81%
Grade III	4063 (14.34%)	12.21%	21.26%	16.60%	16.44%
Grade IV	17 (0.06%)	0.05%	0.06%	0.09%	0%
Unknown grade	16681 (58.86%)	59.11%	56.98%	59.32%	57.79%
Stage					
Localized	16125 (56.90%)	60.27%	48.03%	51.69%	56.20%
Regional	8132 (28.69%)	26.44%	33.33%	32.99%	28.71%
Distant	1865 (6.58%)	5.55%	10.64%	7.47%	6.01%
Unknown/Un-staged	2219 (7.83%)	7.74%	7.99%	7.85%	9.08%
Laterality					
Bilateral, single primary	33 (0.12%)	0.10%	0.12%	0.15%	0.12%
Left Origin of primary	13961 (49.26%)	49.32%	48.76%	49.60%	47.48%
Only One Side is unspecified	87 (0.31%)	0.29%	0.15%	0.48%	0.25%
Paired site No-Inflaterality	617 (2.18%)	2.13%	2.13%	2.01%	2.09%
Right Origin of primary	13643 (48.14%)	48.15%	48.26%	47.76%	50.06%
The primary site of breast cancer					
Nipple	152 (0.54%)	0.60%	0.41%	0.46%	0.25%
The central portion of the breast	1239 (4.37%)	4.45%	3.88%	4.51%	3.80%
Upper-inner quadrant of the breast	2954 (10.42%)	10.51%	9.92%	10.11%	12.76%
Lower inner quadrant of the breast	1422 (5.02%)	5.16%	4.96%	4.63%	4.79%
The upper outer quadrant of the breast	8241 (29.08%)	29.48%	28.70%	28.51%	25.89%
The lower outer quadrant of the breast	1819 (6.42%)	6.58%	6.68%	5.92%	5.28%
The axillary tail of the breast	149 (0.53%)	0.47%	0.76%	0.53%	0.86%
Overlapping lesion of the breast	5843 (20.62%)	20.67%	21.08%	19.90%	22.70%
Breast, not otherwise specified (NOS)	6522 (23.01%)	22.09%	23.62%	25.43%	23.68%
Median Household Income (Missing=1)					
Less than 50,000	2499 (8.82%)	6.23%	3.38%	20.57%	2.58%
50,000-64,999	11694 (41.26%)	40.03%	42.14%	46.43%	27.85%
65,000-74,000	8664 (30.57)	30.49%	40.59%	23.73%	39.51%
75,000 and over	5483 (19.35)	23.24%	13.88%	8.97%	30.06%
Areas where survivors lived (Missing =1)					
Metropolitan areas population>1million	18497 (65.27%)	65.64%	78.16%	53.67%	86.26%
Urban areas 250k<1million	4267 (15.06%)	10.68%	8.17%	33.65%	7.85%
Urban areas Population=250k	2032 (7.17%)	8.65%	6.33%	3.74%	2.33%
Rural areas Adjacent to a metropolitan	2573 (9.08%)	11.06%	5.34%	6.03%	2.45%
Rural areas Not Adjacent to a metropolitan	971 (3.43%)	3.96%	2.01%	2.91%	1.10%

In Texas, the SEER program recorded 18,222 non-Hispanic white patients with breast cancer, 3,429 non-Hispanic black patients, 5,875 Hispanics, and 815 others (American Indian/Alaska Native, Asian or Pacific Islander, and others).

Table 1 shows that White non-Hispanic individuals are much older, with 20.66% aged 75 and older, compared to Black non-Hispanic individuals at 13.36%. Hispanic individuals are also generally younger, with 12.73% aged 75 and older. Others are significantly younger, as only 7.73% of them are 75 and older.

Regarding stage, 60.27% of White non-Hispanic patients were diagnosed with localized-stage disease. In comparison, only 48.03% of Black non-Hispanic patients, 51.69% of Hispanic patients, and 56.20% of other patients received a diagnosis at the localized stage.

Regarding breast subtypes, a notable disparity concerns the subtype HR-/HER2-. Indeed, 18.08% of Black non-Hispanic patients are diagnosed with this subtype compared to only 7.90% of White non-Hispanic patients with the same subtype. When it comes to the negative progesterone receptor, Black non-Hispanic patients lead with 34.47% being diagnosed compared to 26.89% of Hispanic patients, 21.60% of other patients, and only 20.57% of White non-Hispanic patients. Only 29.22% of White non-Hispanic patients received chemotherapy, compared to 42.02% of Black non-Hispanic patients, 39.91% of Hispanic patients, and 38.90% of other patients. 22.23% of all patients received radiation, and 42.53% had systemic therapy or surgery. We also observed that 58.86% of all patients are of unknown grade.

Based on the median household income, 23.24% of White non-Hispanic individuals came from households with an income of $75,000.00 or more, while 13.88% of Black non-Hispanic individuals fell into that income bracket. Additionally, 8.97% of Hispanic patients had a median income of $75,000.00 or more, and 30.06% of other patients were below that level. Furthermore, 6.23% of White non-Hispanic individuals had household incomes below $50,000.00, compared to only 3.38% of Black non-Hispanic individuals and 2.58% of other group patients. However, 20.57% of Hispanic individuals came from households earning less than $50,000.00. These income differences may also have contributed to disparities in survival length.

Kaplan‒Meier survival curves and the log-rank test

The Kaplan-Meier curve is a graphical representation of an estimated survival curve, showing the proportion of observations that have not experienced the event of interest at each time point. In our study, the event of interest is patients dying from causes attributed to breast cancer. We also performed a log-rank test (also known as the Mantel-Cox test), which is a nonparametric test suitable for use when the data are right-censored, as in our study. The log-rank test assesses whether the survival distributions of two or more groups are the same. 

**Figure 1:**
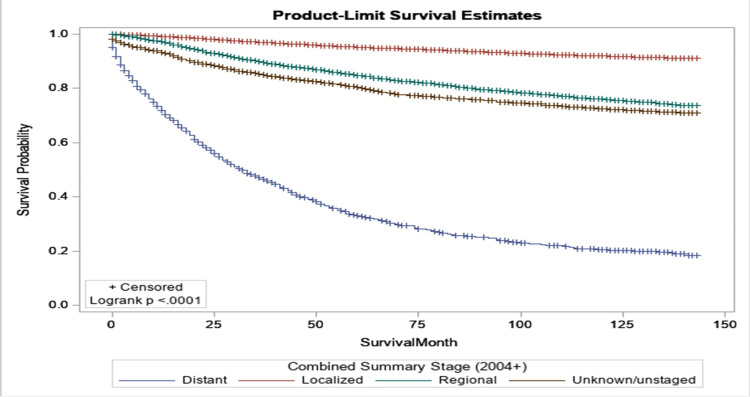
Kaplan-Meier curves by stage for women diagnosed with breast cancer in Texas during 2010 and 2011.

Figure 1 illustrates the relationship between survival probabilities for breast cancer patients and disease stage at diagnosis, as determined by the log-rank test. We found that the stage at diagnosis significantly influences the survival time of breast cancer patients. For example, after 10 years, only 11% of survivors with localized disease have died from breast cancer; in contrast, approximately 80% of survivors with distant stage have died. Therefore, promoting and encouraging early diagnosis is essential for improving the survival prospects of breast cancer patients.

**Figure 2:**
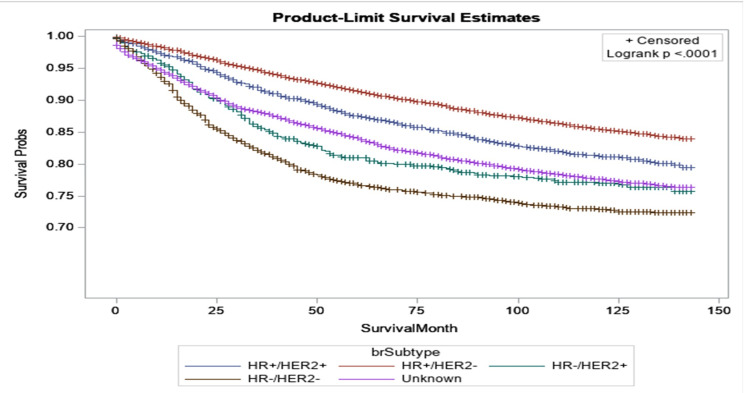
Kaplan-Meier curves by subtype of women diagnosed with breast cancer in 2010 and 2011 in Texas.

Figure 2 shows the relationship between survival probabilities for breast cancer patients and the different subtypes of the cancer at diagnosis. We observed that the subtypes significantly affected patient survival time. For example, after 10 years of survival, roughly 25% of patients with HR-/HER2- died from breast cancer, while only 10% of patients with HR+/HER2-.

**Figure 3:**
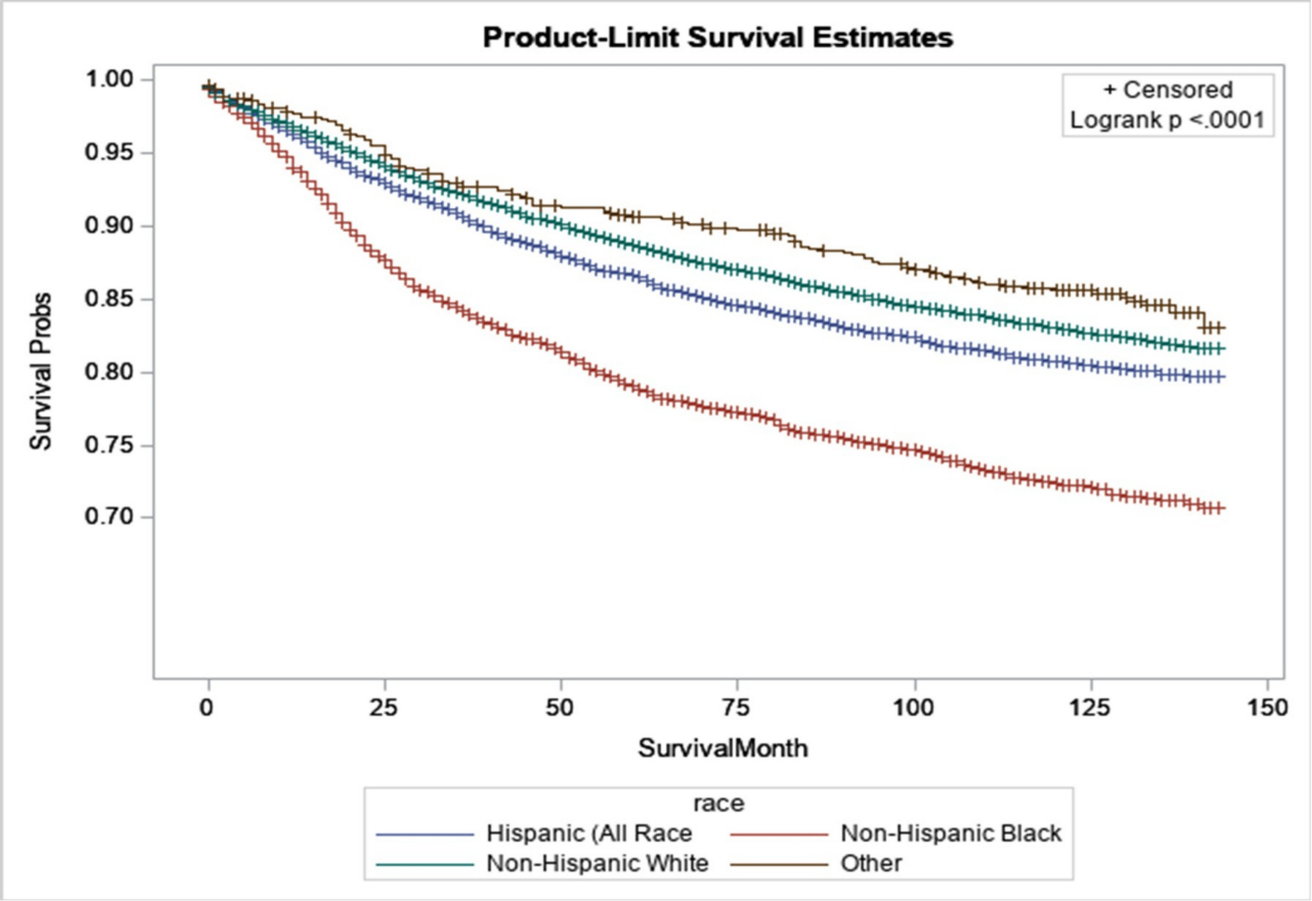
Kaplan-Meier curves stratified by race for women diagnosed with breast cancer in 2010 and 2011 in Texas.

Figure 3 shows the differences in survival probabilities among breast cancer patients based on race and ethnicity. When performing the log-rank test, we observed a statistically significant disparity in survival rates related to race and ethnicity. For example, after 10 years, about 30% of Black non-Hispanic patients had died from breast cancer, approximately 18% of Hispanic patients had died, around 16% of White non-Hispanic individuals had died, and only 10% of the other survivors had died. Therefore, it is crucial to raise awareness about breast cancer in all communities, especially among Black non-Hispanic people.

**Figure 4:**
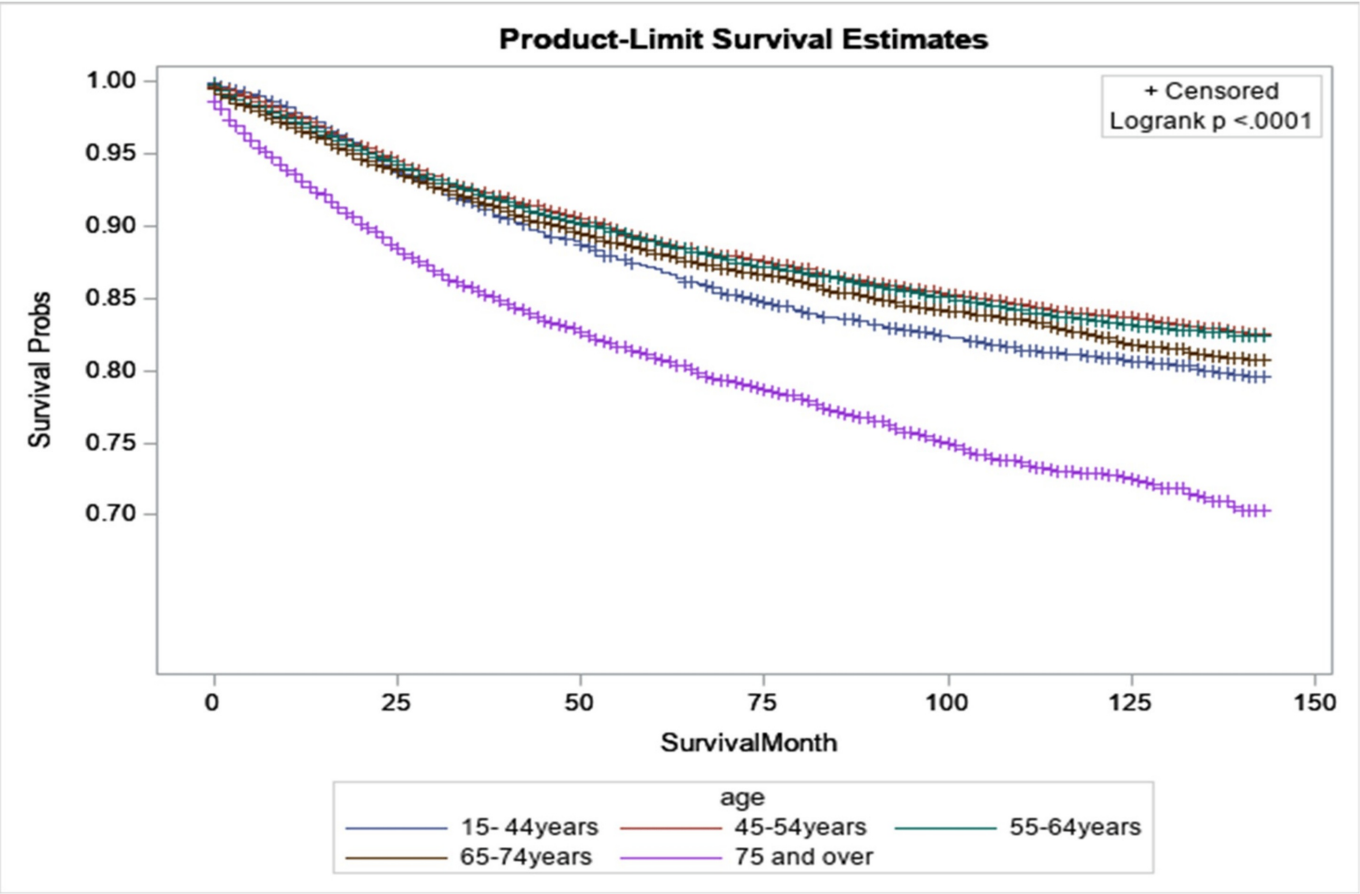
Kaplan-Meier curves by age of women diagnosed with breast cancer in Texas during 2010 and 2011.

Figure 4 shows the relationship between survival probabilities and age for breast cancer patients. Patients diagnosed at 75 years and older had the lowest survival times compared to younger individuals when considering death due to breast cancer. For example, after 10 years, about 30% of patients diagnosed at 75 years and older died from breast cancer, compared to 12% of patients diagnosed between 45 and 54 years old who died from breast cancer.

Finally, the p-values of the log-rank tests for the variable total number of cancers each patient has were more than 0.05, so it was not statistically significant. Therefore, we will exclude this variable from the Cox regression shown in the next section. Furthermore, we ran the stepwise variable selection procedures for Cox regression analysis as well, and this variable (total number of cancers each patient has), along with where the patient lived and the estrogen receptor status, was removed. In the next section, we will present our final Cox proportional hazards regression model.

Non-stratified Cox proportional hazard regression

In this section, we will perform a non-stratified Cox proportional hazards regression analysis of survival time in months, with events defined as mortality due to breast cancer. The hazard function is shown in equation (1). The input variables shown in equation (1) are discussed in Table 1.

$h_i(t)=h_0(t) \exp{\left(\alpha_1 Race_i + \alpha_2 Age_i + \alpha_3 Chemotherapy_i+ \alpha_4 Radiation_i \\+ \alpha_5 SystematicTherapySurgery_i+ \alpha_6 Grade_i+ \alpha_7 Stage_i \\+ \alpha_8 Subtype_i + \alpha_9 Progestone_i+ \alpha_{10} DerivedHER2_i \\ + \alpha_{11} Laterality_i + \alpha_{12} PrimarySite_i+ \alpha_{13} MedianHHIncome_i \right)} \,\,\, (1)$

The outcome of the Cox proportional hazard regression analysis is shown in Table 2. We presented the p-value, the hazard ratio, the 95% CIs, and the parameter estimations. The parameter estimates are for the log hazard ratio for the related variable. Positive parameter estimates indicate a worse prognosis and an increase in the log hazard, or a higher risk, whereas negative parameter values indicate a decrease in the log hazard, a lower risk, and increased survival times. Two asterisks are used to denote statistical significance, which is defined as P-values less than 0.05. Lastly, an elevated hazard is indicated by a hazard ratio larger than 1, and a decreased hazard is indicated by a hazard ratio smaller than 1. Thus, according to Table 2, compared to white non-Hispanic patients with breast cancer, black non-Hispanic patients had a greater hazard ratio (1.383), which indicated that black patients with breast cancer had a 38.3% greater risk of death. Furthermore, compared to survivors aged between 15 and 44 years, survivors aged 75 years and older had a greater hazard ratio (2.368) for death due to breast cancer.

**Table 2:** The results of the non-stratified Cox proportional hazards regression analysis with age in years and household median income in US dollars.

Covariates	Levels	Estimate	Hazard Ratio	95% CI
Race	Reference for race: Non-Hispanic White			
	Hispanics (all races)	-0.01895	0.981	0.912-1.056
	Non-Hispanic Black	0.32408**	1.383	1.281-1.493
	Other	-0.15442	0.983	0.713-1.030
Age (Years)	Reference for age: Less than 44 years old			
	Between 45 and 54	-0.01731	0.983	0.892-1.083
	Between 55 and 64	0.09167	1.096	0.997-1.205
	between 65 and 74	0.29929**	1.349	1.222-1.489
	75 and over	0.86191**	2.368	2.141-2.618
Treatment	Reference for chemotherapy: Chemotherapy (No/Unknown)			
	Chemotherapy (Yes)	0.37029**	1.448	1.338-1.567
	Reference for radiation: Radiation (Yes)			
	Radiation (No/Unknown)	0.14736**	1.159	1.068-1.257
	Reference for Systemic Therapy Surgery: Systemic Therapy Surgery (Yes)			
	Systemic Therapy Surgery (No/Unknown)	0.42835**	1.535	1.416-1.664
Grade	Reference for grade: grade I			
	Grade II	0.50747**	1.661	1.403-1.967
	Grade III	0.80459**	2.236	1.886-2.650
	Grade IV	1.29124**	3.637	1.854-7.136
	Unknown grade	0.56781**	1.764	1.504-2.070
Stage	Reference for stage: Localized			
	Regional	1.14443**	3.141	2.917-3.382
	Distant	2.76439**	15.869	14.566-17.289
	Unknown/Un-staged	1.07052**	2.917	2.603-3.269
Subtype	Reference for subtype: HR+/HER2-			
	HR+/HER2+	-0.55421	0.575	0.250-1.320
	HR-/HER2+	-0.63670	0.529	0.228-1.226
	HR-/HER2-	0.20824**	1.232	1.097-1.382
	Unknown	-0.60663**	0.545	0.313-0.949
Progesterone Status	Reference for Progesterone Status: Positive			
	Borderline/Unknown	0.08601	1.090	0.975-1.226
	Negative	0.37419**	1.454	1.329-1.590
Derived HER2	Reference for derived HER2 Status: Positive			
	Borderline/Unknown	0.16620	1.181	0.633-2.202
	Negative	-0.47366	0.623	0.273-1.421
Laterality	Reference for Laterality: Right Origin of primary			
	Bilateral, single primary	0.16075	1.174	0.745-1.851
	Left Origin of primary	0.00280	1.003	0.947-1.061
	Only One Side-Side unspecific	0.37656**	1.457	1.039-2.044
	Paired Site No Inf laterality	0.39691**	1.487	1.296-1.707
Primary site of the breast cancer	Reference for primary site: Upper-outer quadrant of breast			
	Nipple	0.06890	1.071	0.708-1.620
	Central portion of breast	0.20688**	1.230	1.071-1.413
	Upper-inner quadrant of breast	0.06745**	1.070	0.955-1.199
	Lower inner quadrant of the breast	0.10935	1.116	0.961-1.294
	Lower outer quadrant of the breast	0.02785	1.028	0.902-1.173
	Axillary tail of breast	0.23547	1.266	0.919-1.742
	Overlapping lesion of the breast	0.15066**	1.163	1.067-1.266
	Breast, NOS	0.37550**	1.456	1.346-1.574
Median household income	Reference for median household income: less than $50,000.00			
	50,000 to 64,999	0.06181	1.064	0.962-1.177
	65,000 to 74,999	-0.02568	0.975	0.877-1.083
	75,000 and over	-0.01589	0.984	0.877-1.104

Compared to patients who did not receive chemotherapy, patients who received chemotherapy had a hazard ratio of 1.448. Individuals without systemic therapy surgery had a greater hazard ratio (1.535) than individuals who underwent the procedure.

Compared to survivors diagnosed with Grade I, those with more advanced grades are more likely to die early. For example, Grade III has a hazard ratio of 2.236, indicating a 123.6% higher risk of death. Additionally, survivors with more advanced-stage breast cancer are more likely to die early compared to those diagnosed with localized-stage breast cancer. For instance, survivors with distant stage breast cancer have a hazard ratio of 15.869, meaning they have 15.869 times the risk of dying at any given time compared to those with localized stage. Early diagnosis is crucial for improving survival rates.

Compared to patients who were diagnosed with HR+/HER2- subtype, patients with HR-/HER2- have a hazard ratio of 1.232. Patients with negative progesterone have a hazard ratio of 1.454 compared to those with positive progesterone. Individuals with only one side-side unspecific had a hazard ratio of 1.457 when compared to individuals with the right origin of primary laterality.

Compared to individuals with the upper-outer quadrant of the breast as the primary site, those with overlapping lesions had a hazard ratio of 1.163. Additionally, survivors with breasts or NOS had a hazard ratio of 1.456. These findings suggest that survivors with overlapping lesions and survivors with breasts not otherwise specified (NOS) are more likely to die earlier than those with lesions in the upper-outer quadrant.

Therefore, among the top prognostic factors in our database are White non-Hispanic patients aged 45 to 54 diagnosed with localized stage and grade one tumors, with the primary site in the upper-outer quadrant of the breast, right-sided primary laterality, and a subtype indicating the presence of HR and absence of HER2, i.e., HR+/HER2-. These patients also underwent systemic therapy surgery, did not receive chemotherapy, tested positive for progesterone, and had a household income of $75,000.00 or more.

Black non-Hispanic patients aged 75 and older diagnosed with distant stage, grade IV cancer, with the primary site unspecified, only one-sided unspecific laterality, and an HR-/HER2-negative subtype, who received chemotherapy but did not have surgery as systemic therapy, tested negative for progesterone, and had a household income of less than $50,000.00 are among the worst prognostic factors identified in our database.

Random survival forests

Random Survival Forests for Classification

**Figure 5:**
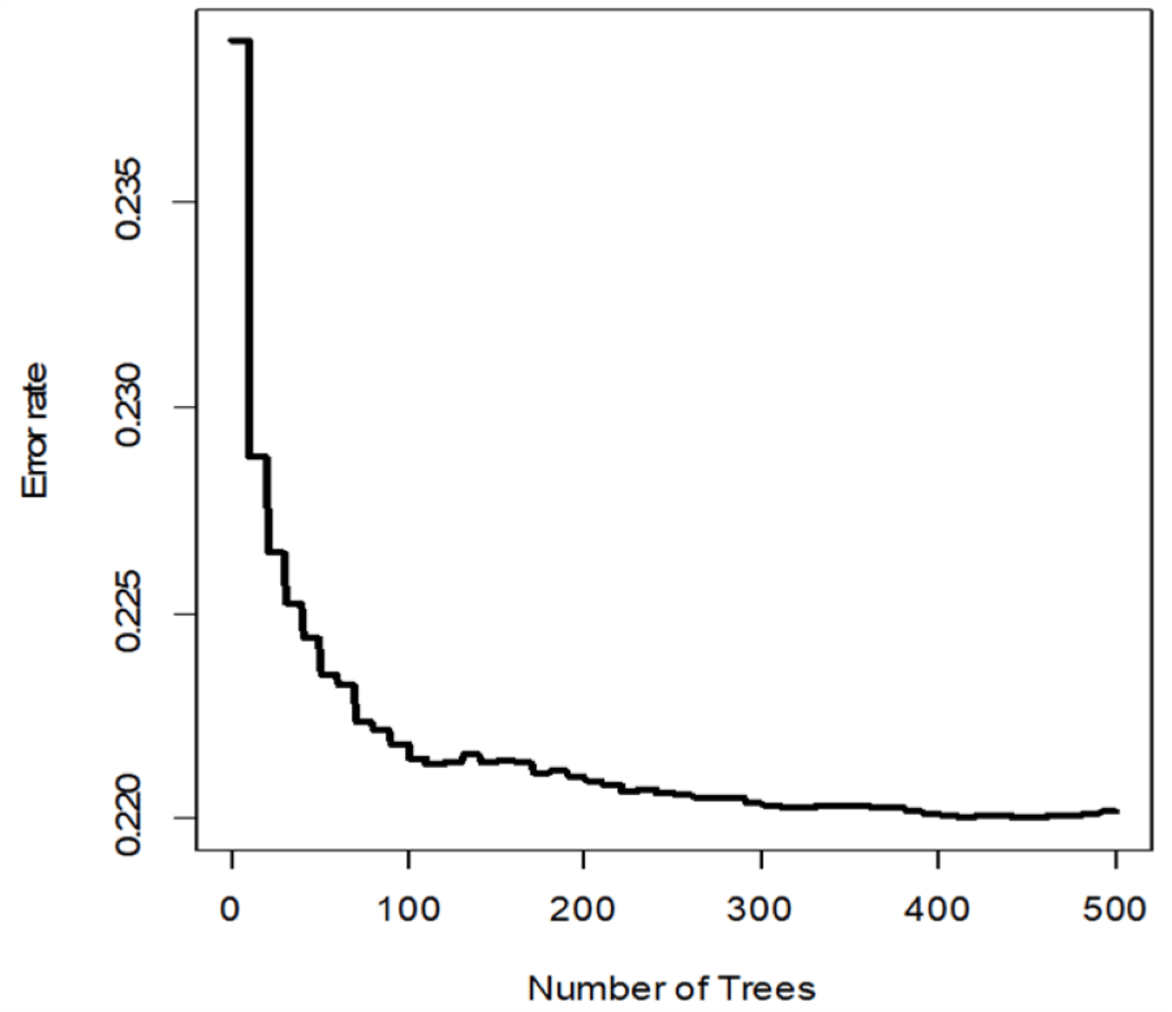
Error rate decreases as the number of trees increases.

The Random Survival Forest (RSF) is an extension of the Random Forests approach for studying right-censored survival data. It excels at handling high-dimensional data and allows complex interaction effects between covariates without requiring proportional risks. The number of trees, the number of variables to possibly split at each node (mtry), and the number of random splits for splitting a variable (nsplit) are important hyperparameters. In our model, we used 1000 trees, the mtry was equal to four and the nsplit was three. Figure 5 displays an error rate of only 0.22.

**Figure 6:**
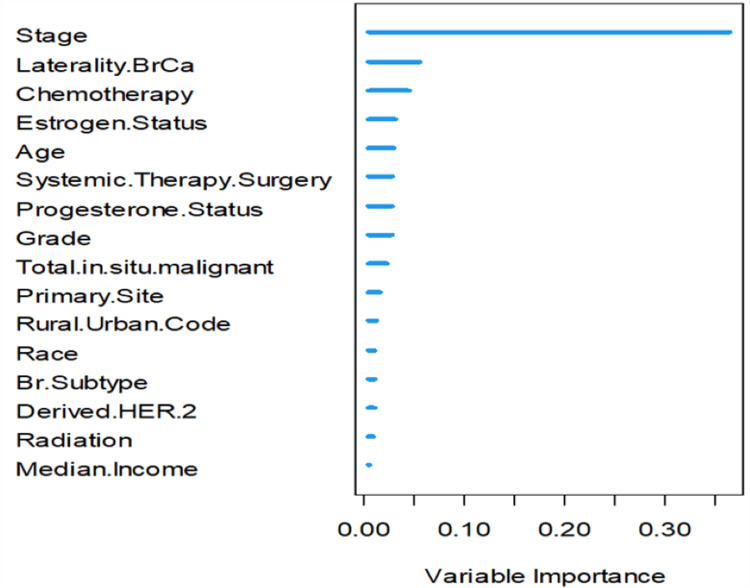
Feature importance in predicting breast cancer patients' survival using the RSF model. RSF: Random Survival Forest

Moreover, Figure 6 shows the importance of the variables in the trained RSF model. The RSF model for breast cancer patients’ survival reveals that stage is once again the most important predictor of breast cancer survival length. The stage at diagnosis contributes 36.16% to the overall prediction of survival length. Breast cancer laterality, which is the second most important variable, only contributes 5.55% to the overall prediction of survival length. Therefore, early detection is essential in predicting the survival of breast cancer patients.

Predictions Using the Random Survival Forests

**Figure 7:**
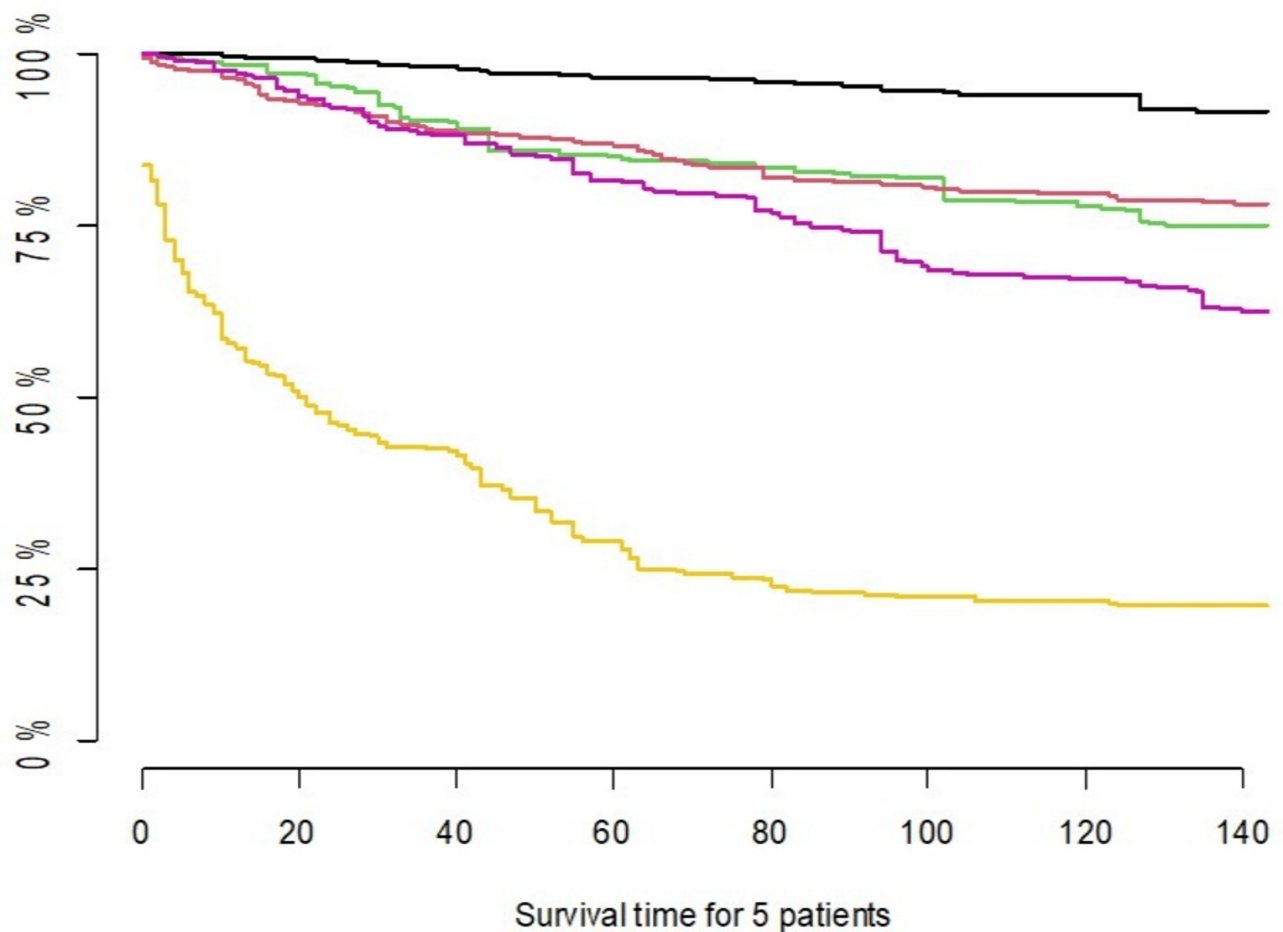
Predicted survival curves for five randomly selected patients.

After training the RSF model, we will use it to make predictions. The model's ability to distinguish between patients with different survival histories and rank their expected survival times is assessed using the C-index statistic, which ranges from 0 to 1. A higher C-index indicates better discriminative ability. In our model, the C-index is 0.7665, demonstrating good risk discrimination. Figure 7 displays predicted survival curves for five randomly selected patients.

Average Treatment Effect Using Mahalanobis Distance Matching

To understand the role of stage at diagnosis as the most important variable in prediction for the random survival forest and its statistical significance in both stratified and non-stratified Cox proportional hazards regressions, we perform causal inference for localized stage at diagnosis versus distant stage. We estimated the average treatment effect, which is the difference in mean outcome (survival length) between patients with localized or distant cancer at diagnosis. The average treatment effect gauges the causal impact of being diagnosed with localized versus distant breast cancer on survival duration. Additionally, we performed the Wilcoxon test, which revealed a significant difference between the two matched groups with a p-value of less than 2.2e-16. Distributions of variables before and after matching, using Mahalanobis distance, support the success of the matching process, as shown in Table 3.

**Table 3:** Balanced numbers in both groups of treatment after matching.

Covariate	Levels	Before Matching				After Matching			
		Localized Stage		Distant Stage		Localized Stage		Distant Stage	
		n	%	n	%	n	%	n	%
Race	Non-Hispanic White	10983	68.11	1012	54.26	922	53.51	922	53.51
	Non-Hispanic Black	1647	10.21	365	19.57	346	20.08	346	20.08
	Hispanic (All Races)	3037	18.83	439	23.54	408	23.68	408	23.68
	(other)	458	2.84	49	2.63	47	2.73	47	2.73
Laterality	Bilateral, single primary	1	0.0062	13	0.697	0	0	0	0
	Left Origin of primary	8107	50.28	892	47.83	891	51.71	891	51.71
	Only One Side is unspecified	21	0.13	16	0.86	8	0.46	8	0.46
	Paired site No-Inf laterality	4	0.024	124	6.65	4	0.23	4	0.23
	Right Origin of primary	7992	49.56	820	43.97	820	47.59	820	47.59
Age (Years)	15-44	1708	10.59	256	13.72	248	14.39	248	14.39
	45-54	3293	20.42	404	21.66	384	22.29	384	22.29
	55-64	4246	26.33	501	26.86	470	27.28	470	27.28
	65-74	3830	23.75	367	19.68	322	18.69	322	18.69
	75 and over	3048	18.90	337	18.07	299	17.35	299	17.35
Chemotherapy	Yes	3524	21.85	1000	53.62	950	55.14	950	55.14
	No/unknown	12601	78.15	865	46.38	773	44.86	773	44.86

**Figure 8:**
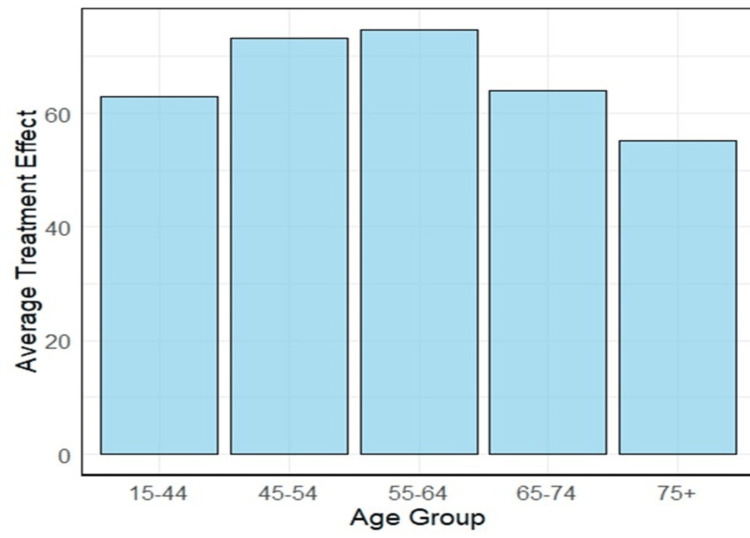
The average treatment effect of localized versus distant stage at diagnosis (measured in months), shown by age group.

We found that the average extended lifetime of patients diagnosed with the localized stage was about 67.34 months, compared to patients diagnosed with the distant stage. These averages vary across age groups (see Figure 8). For example, the average extended lifetime of patients diagnosed between the ages of 55 and 64 with the localized stage was about 74.70 months, compared to patients diagnosed with the distant stage in the same age group. However, the average extended lifetime of patients diagnosed at ages 75 and older with the localized stage was only about 55.28 months, compared to patients diagnosed with the distant stage in the same age group.

Among the best prognostic factors identified in our database are White non-Hispanic patients aged 45 to 54 diagnosed with localized stage and grade one cancer. The primary tumor site is the upper-outer quadrant of the breast, with right-side laterality, and a subtype of HR+/HER2-. These patients also underwent systemic therapy surgery, did not receive chemotherapy, tested positive for progesterone, and had a household income of $75,000.00 or more.

## Discussion

This study enabled us to examine the primary factors that may influence the survival duration of breast cancer patients in Texas by analyzing an 11-year retrospective cohort dataset. According to the Texas Department of State Health Services, breast cancer is the most frequently diagnosed cancer and the second leading cause of cancer-related deaths among Texan women, highlighting the importance of this topic.

Our research shows that being diagnosed with breast cancer at the most advanced stage is the main cause of death from the disease. These findings align with earlier studies on breast cancer. According to Giaquinto et al. (2024), the 5-year relative survival rate ranges from over 99% for localized stage illness to 87% for regional stage disease and 32% for distant stage disease, highlighting that the stage at diagnosis is the strongest predictor of prognosis [22].

Furthermore, our paper shows that, compared to women of other races and ethnicities, Black women are diagnosed at a later stage. Black non-Hispanic individuals are diagnosed at a distant stage at a rate of 10.64%. In comparison, White non-Hispanic individuals are diagnosed at a rate of 5.55%, Hispanic individuals at 7.47%, and others at 6.01%. The triple negative breast cancer subtype (HR-/HER2-) is also diagnosed in 18.08% of Black women, compared to 7.90% of non-Hispanic White women, 11.34% of Hispanic women, and 6.99% of other women. These factors may play a significant role in explaining why Black non-Hispanic women have the lowest breast cancer survival rates. Other studies in the literature also indicate that Black non-Hispanic women have the lowest survival rate [29].

Cancer patients' income or neighborhood may also influence their chances of survival. Even after adjusting for stage at diagnosis, cancer treatment, and individual lifestyle factors, living in highly deprived neighborhoods was associated with a statistically significant 47% increased risk of dying from breast cancer, according to a cohort study of 2290 Black women with the disease conducted [30].

Our study also reveals that White non-Hispanics are being diagnosed with breast cancer at an older age compared to other races or ethnicities. This study provides us with a better understanding of how socioeconomic status, illness characteristics, and various treatment parameters affect the survival of patients with breast cancer. It is imperative that we continue to advocate for early screening and develop targeted interventions to reduce health disparities.

There are several limitations to this retrospective study. If we had access to additional survivor data, such as patient education levels, health behaviors, and comorbidities, we believe this analysis would be more robust.

## Conclusions

This study highlights the importance of early diagnosis and screening for women in Texas. Doctors continue to search for more effective treatment plans for breast cancer survivors, but they need more funding. Therefore, our society must continue to prioritize cancer research. In our future research, we will incorporate genetic mutations and provide more detailed information about treatment regimens, including the number of chemotherapy rounds each patient received. Additionally, we will continue to investigate disparities in survival among groups such as women under 44 years old, women with distant-stage cancer, Black women, and patients with triple-negative breast cancer. Our research can help policymakers develop health policies that target vulnerable populations to improve health outcomes for breast cancer patients.
